# Effect of a Combination of Omeprazole Plus Sustained Release Baclofen Versus Omeprazole Alone on Symptoms of Patients with Gastroesophageal Reflux Disease (GERD)

**Published:** 2014

**Authors:** Mohammad Abbasinazari, Yunes Panahi, Seyed Alireza Mortazavi, Fanak Fahimi, Ghasem Valizadegan, Reza Mohtashami, Mohammad Amin Pourhoseingholi, Kambiz Shirvani Bakhtiari

**Affiliations:** a *Department of Clinical Pharmacy, Shahid Beheshti University of Medical Sciences, Tehran, Iran*; b *Chemical Injuries Research Center, Baqiyatallah University of Medical Sciences, Tehran, Iran.*; c *Department of Pharmaceutics, Shahid Beheshti University of Medical Sciences, Tehran, Iran.*; d *Baqiyatallah Research Center for Gastroentrology and Liver Diseases, Tehran, Iran.*; e *Medicine Quran& Health Research Center, Baqiyatallah University of Medical Sciences, Tehran, Iran.*; f *Research Center for Gastroenterology and Liver Diseases, Shahid Beheshti University of Medical Sciences, Tehran, Iran.*

**Keywords:** Baclofen, Omeprazole, GERD, Sustained release

## Abstract

Previous studies have reported the efficacy of baclofen in the treatment of Gastroesophageal Reflux Diseases (GERD). The objective of present study is to evaluate the effect of co-administration of omeprazole 20 mg/d plus sustained Release baclofen (SR baclofen) vs. omeprazole 20 mg/d plus placebo on alleviation of symptoms in patients with a diagnosis of GERD. A prospective, double blind, placebo controlled trial included 60 patients with diagnosis of GERD have been done. Patients were randomly selected to receive either SR baclofen or a placebo in addition to omeprazole 20 mg/d for a period of 2 weeks. Patients were questioned regarding heartburn, regurgitation, chest pain and hoarseness at the base line and after 2 weeks. All patients tolerated the medications and no patients failed to complete the study due to adverse drug reactions. A total of 53 patients completed the study, 25 in SR baclofen and 28 in placebo group. After 2 weeks, 1 patient (4%) in SR baclofen group reported heartburn and regurgitation. However 13(46.4%) and 15 (53.6%) of patients in the placebo group had heartburn and regurgitation respectively. The analysis of the data shows that there is a significant difference between the two groups in heartburn and regurgitation (p < 0.0001, p < 0.0001 respectively). Statistical analysis revealed a significant difference in two groups regarding total GERD score (p <0.0001). The results of the present study suggest that a combination of SR baclofen and omeprazole may be a more effective treatment for heartburn and regurgitation than omeprazole alone

## Introduction

The passage of gastric contents into the oesophagus is a normal physiologic phenomenon. Gastroesophageal reflux disease (GERD) is defined as a condition that develops when the reflux of stomach content into the oesophagus causes troublesome symptoms or complications (1). Although mortality associated with GERD is rare, it has direct impact on the quality of life (QoL). GERD patients report a lower QoL than healthy individuals, especially in those with nighttime GERD (2). Shaker *et al.* have reported that 78% of GERD patients presented with nocturnal symptoms and 63% of those patients reported that sleep was negatively affected (3). GERD-related impairment of QoL is known to be similar to the reduction in QoL reported by patients with diabetes or cancer (4).The true prevalence of GERD is unknown because there is no gold standard for diagnosis. The diagnosis of GERD is made using some combination of symptom presentation, objective testing with endoscopy, ambulatory reflux monitoring, and response to antisecretory agents (5).

In most cases, treatment for GERD involves two important modalities: lifestyle changes and pharmacologic intervention, primarily with acid suppressing agents (6). In some cases, anti-reflux surgery or endoscopic therapies may be considered (7). Proton pump inhibitors (PPIs) are the current cornerstone of pharmacological GERD treatment. PPIs require several days to achieve maximum suppression of gastric acid, which is a disadvantage if the drugs are needed for intermittent or short-term use only when symptoms occur. Another problem is that PPIs only target the acid component, not the non-acid component that also contributes to reflux (8, 9). It has been suggested that Gama Amino Butyric Acid (GABA), a neurotransmitter, may be implicated in transient, lower oesophageal sphincter relaxations (TLESRs) (10). GABA is a fast-acting inhibitory neurotransmitter in the mammalian brain that affects different subtypes, including GABA-A and GABA-B receptors (11).

Previous studies have reported that treatment of GERD with immediate release baclofen, a GABA-B agonist, is effective. In two studies that evaluated the effect of baclofen on TLESRs in humans, results showed a reduction in the rate of post prandial TLESRs and acid reflux episodes per hour in healthy volunteers and in patients with reflux oesophagitis (10, 12). In another study Ciccaglione and Marzio evaluated the effect of acute and chronic administration of baclofen on 24-hour pH-metry and symptoms in control subjects and in patients with GERD. Results showed that baclofen reduces 24 hour reflux and increases gastric pH in both GERD patients and controls (13).

Because the half-life of baclofen is short (between 4.5 and 6.8 hours) in healthy subjects (14), it has been administered 3–4 times per day in previous studies. This could be the cause of observed low adherence of patients to baclofen regimens. PPIs such as omeprazole are usually the first choice in the treatment of GERD (9), but they are sometimes insufficient in reliving symptoms of GERD. The aim of the present study is to evaluate the effect of co-administration of omeprazole 20 mg/d plus SR baclofen vs. omeprazole 20 mg/d plus placebo on alleviation of symptoms in patients with a diagnosis of GERD.

## Experimental

A double-blind, placebo-controlled study design was employed and the proposal of the study was approved by the ethical committee of the Shahid Beheshti University of Medical Sciences. Also, it was registered in Australian New Zealand clinical trial registry site with number ACTRN12613000910707. Between March 2013 and December 2013, a clinical trial was conducted on patients who referred to the gastrointestinal clinic of Baqiyatallah Hospital with a diagnosis of GERD. The criteria for diagnosis of GERD were based on symptoms and the use of the Persian validated Mayo Gastroesophageal Reflux Questionnaire (MGEQ). Mayo-GERQ is one of the most widely used questionnaires for primary diagnosis of GERD. It is self-administered and consists of 80 questions that measure symptoms during the prior year and provide a total medical history regarding GERD. This questionnaire has been shown to be reliable (median kappa: 0.70) on retest and valid (median kappa, 0.62) in comparison to physician interview (15). Nasei-Moghaddam *et al. *have translated it into Persian and validated it in a Persian population (16). Informed consent was obtained from patients with diagnosis of GERD who agreed to enroll in the study after a thorough explanation of the aims, risks, and benefits of this study.

Patients were excluded if they had acute symptoms requiring endoscopy, evidence of significant hepatic and/or renal diseases, seizures, neurological disorders, or intolerance to baclofen. Patients who were taking medications which alter LES sphincter function were asked to stop therapy one week prior to enrollment in this study. All enrolled patients received omeprazole 20 mg/d. In addition, each patient randomly received SR baclofen tablet (10 mg) twice daily or a placebo. Patients were randomized using a computer-generated scheme to receive either placebo or SR baclofen tablet.

Patients were asked to take the medications for 2 weeks and then return to the clinic for follow up. Before treatment and at the end of the two weeks, each patient was questioned about GERD symptoms. Patients were asked to report the presence of four symptoms, including heartburn, acid regurgitation, chest pain, and hoarseness. For each symptom, the presence of that condition resulted in a score of one, while absence of the symptom scored zero. The total GERD score is defined as the sum of the scores for all four symptoms. Data were analyzed with SPSS-12 and a p-value of less than 0.05 was considered statistically significant.

## Results

**Figure 1:**
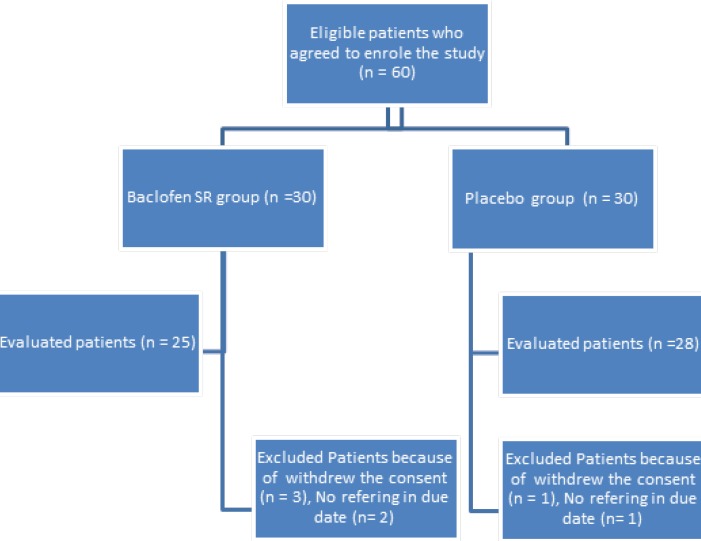
Flowchart of patients

A total of 60 patients were enrolled in the study. Seven patients were eliminated from the study due to withdrawal of consent or not reporting for follow up. The flow of the participants through the study is shown in Figure 1. A total of 53 patients completed the study, 25 in the SR baclofen group and 28 in the placebo group. Baseline characteristics of the patients are shown in Table 1. The two groups were comparable at baseline with respect to demographic characteristics such as age, sex, Body Mass Index (BMI), smoking habit, and marital status. The two groups were also similar in the percentage of existence of baseline symptoms of GERD, including heartburn, regurgitation, chest pain and hoarseness, and the total GERD scores were not statistically different in the two groups at baseline (p = 0.87).

**Table 1:** Demographic and bseline GERD symptoms in studied patients

**Parameter**	**SR baclofen group** **(n = 25)**	**Placebo group** **(n = 28)**	**p-value**
Age(years)	41.0 ± 14.5	36.8 ± 9.0	0.2
Male Gender (%)	11 (44%)	13(46.4%)	0.86
Smokers (%)	2 (8%)	2 (7.1%)	0.9
Married patients (%)	23(92%)	25 (89.3%)	0.73
BMI (kg/m^2^)	26 ± 4.0	25.5 ± 4.9	0.69
Patients with Heartburn (%)	21(92%)	23(75%)	0.05
Patients with regurgitation (%)	19(76%)	23(82.1%)	0.84
Patients with Chest pain (%)	7(28%)	10(35.7%)	0.09
Patients with Hoarseness (%)	3(12%)	3(10.7%)	0.67
Total GERD score	2.1 ± 0.8	2.0 ± 0.8	0.71

After 2 weeks, the four symptoms of GERD assessed again by questioning patients. Table 2 shows the number of patients with each symptom and the total GERD scores. Analysis of the data indicates a significant difference between the two groups in the prevalence of heartburn (p < 0.0001) and regurgitation (p < 0.0001), whereas there is no significant difference in chest pain (p = 0.35) or hoarseness (p = 0.93). The average total GERD score was 1.1 ± 1.0 in the placebo group versus 1.6 ± 0.8 in the SR baclofen group.

Statistical analysis revealed a significant difference in total GERD score (p < 0.0001) between the two groups. All patients tolerated the medications and no patients failed to complete the study due to adverse drug reactions.

**Table 2:** GERD symptoms of patients after 2 weeks

	**SR baclofen group** **(n = 25)**	**Placebo group** **(n = 28)**	**p-value**
Patients with Heartburn (%)	1 (4%)	13 (46.4%)	< 0.0001
Patients with regurgitation (%)	1 (4%)	15 (53.6%)	< 0.0001
Patients with Chest pain (%)	1 (4%)	3 (10.7%)	0.35
Patients with Hoarseness (%)	1 (4%)	1 (3.6%)	0.93

## Discussion

For the first time Lidums *et al.* have shown that baclofen decreases reflux events and acid in normal individuals and in patients with GERD. Unlike PPIs, which work by inhibiting acid secretion, baclofen reduces reflux by inhibiting transient relaxations of the LES, which are the primary cause of reflux events (17).

Sampat *et al. *have reported that sustained release baclofen are efficacious, convenient and better tolerated alternative to immediate release baclofen in patients with neurogenic spasticity (18). There is not any report regarding effect of SR baclofen on control of GERD in the literature. The purpose of this study was to compare the effect of a 2 week treatment with SR baclofen plus omeprazole versus omeprazole alone on GERD symptoms. Previous studies have been done with immediate release baclofen, but we have used SR baclofen, which requires less frequent dosages, for patient convenience. Because the effect of SR baclofen on GERD symptoms was previously unknown, all patients received omeprazole, one of the first line drugs used for pharmacotherapy of GERD. There is an overlap between symptoms of Non-ulcer dyspepsia (NUD) and GERD (19), and we have used a validated questionnaire to assure accurate diagnosis of GERD (16). Also our study was matched for demographic parameters such as BMI and smoking habits between the two groups. Elevated BMI and smoking are two risk factors for development and aggravation of GERD symptoms. The exact mechanism by which elevated BMI causes reflux disease still remains to be clearly defined, but several studies have addressed the potential relationship between GERD and elevated BMI (20). Smith *et al*. have reported that cigarette smoking is known to affect adversely the defense mechanisms against reflux of acid gastric contents into the esophagus (21). In addition, there was no significant difference between the two groups with respect to GERD symptoms at the baseline of the study. Xu *et al.* have evaluated the efficacy and safety of baclofen 20 mg trice daily in addition to omeprazole 20 mg/d for treatment of refractory GERD induced chronic cough unresponsive to standard therapy. In their study, reflux symptoms were scored by a Chinese version of GERD diagnostic questionnaire. After 8 weeks, the reflux symptoms score decreased significantly from 8.0 ± 1.6 to 6.8 ± 0.8 (p = 0.023) (22). Like our study, adverse effects were usually tolerable and waned within 1-3 weeks in Xu *et al.* study. 

 As reported by Cossentino *et al.* (23), our results suggest that, after two weeks, the percentage of patients with regurgitation and total GERD symptoms significantly decreased in the SR baclofen plus omeprazole group versus the group given omeprazole alone. Contrary to our results, they did not see any change in the percentage of heartburn in the baclofen group. Some of the differences between our results and those of Cossentino *et al*. are related to different study design. They administered immediate release baclofen or a placebo and we administered omeprazole 20 mg/d in addition in both groups. Ciccaglione *et al.* have evaluated the effect of chronic administration of immediate release baclofen, 10 mg four times per day versus a placebo for control of GERD symptoms. They reported that the intensity and frequency of total symptoms, such as heartburn and regurgitation, significantly improved after treatment with baclofen in all patients, while there was no change in total symptom scores with a placebo (13). Drowsiness is the most important adverse drug reaction associated with baclofen. Both in our study and in that of Cossentino *et al*. drowsiness did not limit baclofen use. It seems that the side effects of baclofen are not a limiting factor for its use in the treatment of GERD. Also it seems that baclofen may be effective in quality of life parameters. Orr *et al*. have suggested that baclofen could therefore be considered as a useful adjunct therapy to PPIs in patients with nighttime heartburn and sleep disturbance who continue to have heartburn and/or sleep complaints despite PPI therapy (24). We have not evaluated quality of life in our patients and in future we recommend evaluation of quality of life in patients receiving SR baclofen for treatment of GERD.

Unlike the study of Cossentino *et al*., we have not administered gastrointestinal procedures such as pH-metery and oesophageal manometry. This could be a limitation for interpretation of results from our study. Cossentino *et al*. reported that baclofen was associated with an important decrease in the percentage of upright reflux shown by 24-hour pH monitoring. Although we did not do pH-metry or oesophageal manometry in study subjects, these measurements should be included in future research. Given the insufficient response in some patients to PPI monotherapy, the results of the present study suggest that a combination of SR baclofen and omeprazole may be a more effective treatment for heartburn and regurgitation than omeprazole alone

## References

[1] Vakil N, Van Zanten SV, Kahrilas P, Dent J, Jones R (2006). The Montreal definition and classification of gastroesophageal reflux disease: a global evidence-based consensus. Am. J. Gastroenterol.

[2] Farup C, Kleinman C, Sloan S, Ganoczy D, Chee E, Lee C, Revicki D (2001). The impact of nocturnal symptoms associated with gastroesophageal reflux disease on health-related quality of life. Arch. Int. Med.

[3] Shaker R, Castell DO, Schoenfeld PS, Spechler SJ (2003). Nighttime heartburn is an under-appreciated clinical problem that impacts sleep and daytime function: the results of a Gallup survey conducted on behalf of the American Gastroenterological Association. Am. J. Gatroenterol.

[4] Jung SH, Oh JH, Jie BS, Oh SH, Kim JS, Jeon JS, Choi MG (2012). Typical symptoms rather than extraesophageal symptoms affect the quality of life in gastroesophageal reflux disease. Turk. J. Gastroenterol.

[5] Katz PO, Gerson LB, Vela MF (2013). Guidelines for the diagnosis and management of gastroesophageal reflux diseases. Am. J. Gastroenterol.

[6] Chait MM (2010). Gastroesophageal reflux disease: Important considerations for the older patients. World J. Gastrointest Endosc.

[7] Allen JI (2012). Endoscopy for gastroesophageal reflux diserase:choosewisely. Ann. Int. Med.

[8] Tack J (2010). Emerging medical therapies for treatment of GERD. Gastroenterol. Hepatol.

[9] Wang YK, Hsu WH, Wang SS, Lu CY, Kuo FC, Su YC, Yang SF, Chen CY, Wu DC, Kuo CH (2013). Current Pharmacological Management of Gastroesophageal Reflux Disease. Gastroenterol. Res. Pract.

[10] Vela MF, Tutuian R, Katz PO, Castell DO (2003). Baclofen decreases acid and non-acid post-prandial gastro-oesophageal reflux measured by combined multichannel intraluminal impedance and pH. Aliment. Pharmacol. Ther.

[11] Afarineshe Khaki MR, Pahlavan Y, Sepehri G, Sheibani V, Pahlavan B (2013). Antinociceptive effect of aqueous extract of origanum vulgare l. in male rats: possible involvement of the GABAergic system. Iran. J. Pharm. Res.

[12] Van Herwaarden MA, Samsom M, Rydholm H, Smout AJ (2002). The effect of baclofen on gastro-oesophageal reflux, lower oesophageal sphincter function and reflux symptoms in patients with reflux disease. Aliment. Pharmacol. Ther.

[13] Ciccaglione AF, Marzio L (2003). Effect of acute and chronic administration of the GABA B agonist baclofen on 24 hour pH metry and symptoms in control subjects and in patients with gastro-oesophageal reflux disease. Gut.

[14] Dias LS, Vivek G, Manthappa M, Acharya RV (2011). Role of hemodialysis in baclofen overdose with normal renal function. Indian J. Pharmacol.

[15] Locke GR, Talley NJ, Weaver Al, Zinsmeister (1994). A new questionnaire for gastroesophageal reflux disease. Mayo. Clin. Proc.

[16] Nasseri-Moghaddam S, Razjouyan H, Habibi R, Rafaat-Zand K, Ahrari B, Nouraie M, Majdzadeh R, Vahedi H, Malekzadeh R (2008). Reliability, validity, and feasibility of the mayo gastro-esophageal reflux questionnaire (GERQ) in a persian-speaking population. Iran. J. Pub. Health.

[17] Lidums I, Lehmann A, Checklin H, Dent J, Holloway RH (2000). Control of transient lower esophageal sphincter relaxations and reflux by the GABA B agonist baclofen in normal subjects. Gastroenterol.

[18] Sampat NG, Kulkarni RV, Sase N, Joshi NH, Vora PB, Bhattacharya AK, Lakhani J D, Bhowmik SS (2009). Once daily baclofen sustained release or gastro-retentive system are acceptable alternatives to thrice daily baclofen immediate release at same daily dosage in patients. Neurol India.

[19] Choung RS, Locke GR, Schleck CD, Zinsmeister AR, Talley NJ (2012). Overlap of dyspepsia and gastroesophageal reflux in the general population: one disease or distinct entities?. Neurogastroenterol. Motil.

[20] Emerenziani S, Rescio MP, Guarino MP, Cicala M (2013). Gastro-esophageal reflux disease and obesity, where is the link?. World J. Gastroenterol.

[21] Smit CF, Copper MP, van Leeuwen JA, Schoots IG, Stanojcic LD (2001). Effect of cigarette smoking on gastropharyngeal and gastroesophageal reflux. Ann. Otol. Rhinol. Laryngol.

[22] Xiang-Huai Xu, Zhong-Min Yang, Qiang Chen, Li Yu, Si-Wei Liang, Han-Jing Lv, Zhong-Min Qiu (2013). Therapeutic efficacy of baclofen in refractory gastrointestinal reflux-induced cough. World J. Gastroenterol.

[23] Cossentino MJ, Mann K, Armbruster SP, Lake JM, Maydonovitch C, Wong RK (2012). Randomised clinical trial: the effect of baclofen in patients with gastro-oesophageal reflux - a randomised prospective study. Aliment. Pharmacol. Ther.

[24] Orr WC, Goodrich S, Wright S, Shephred K, Mellow M (2012). The effect of baclofen on nocturnal gastroesophageal reflux and measures of sleep quality: a randomized,cross-over trial. Neurogastroenterol. Motil.

